# The transcriptional portraits of the neural crest at the individual cell level

**DOI:** 10.1016/j.semcdb.2022.02.017

**Published:** 2022-03-05

**Authors:** Alek G. Erickson, Polina Kameneva, Igor Adameyko

**Affiliations:** a Department of Physiology and Pharmacology, Karolinska Institutet, 17165 Stockholm, Sweden; b Department of Neuroimmunology, Center for Brain Research, Medical University Vienna, 1090 Vienna, Austria

**Keywords:** Neural crest, Multipotency, Single cell transcriptomics, Computational biology, Fate determination, Micro-heterogeneity, Fate decisions

## Abstract

Since the discovery of this cell population by His in 1850, the neural crest has been under intense study for its important role during vertebrate development. Much has been learned about the function and regulation of neural crest cell differentiation, and as a result, the neural crest has become a key model system for stem cell biology in general. The experiments performed in embryology, genetics, and cell biology in the last 150 years in the neural crest field has given rise to several big questions that have been debated intensely for many years: “How does positional information impact developmental potential? Are neural crest cells individually multipotent or a mixed population of committed progenitors? What are the key factors that regulate the acquisition of stem cell identity, and how does a stem cell decide to differentiate towards one cell fate versus another?” Recently, a marriage between single cell multi-omics, statistical modeling, and developmental biology has shed a substantial amount of light on these questions, and has paved a clear path for future researchers in the field.

## 1. The brief history of the neural crest research and emerging questions

Neural crest, often referred to as the fourth germ layer, is a multipotent embryonic population of progenitors giving rise to a large array of cell types during development. Since the invention of fate mapping technologies, the overall developmental repertoire of the neural crest lineage has been well-established. This long list includes melanocytes, peripheral sensory neurons, autonomic nervous system derivatives such as parasympathetic and sympathetic neurons, chromaffin cells of the adrenal medulla, enteric neurons, peripheral glia and other nerve-associated cells (Schwann cells, satellite and enteric glial cells, endoneurial fibroblasts). In addition to this profound spectrum, the cranial neural crest gives rise to ectomesenchymal derivatives in the face, neck and teeth (mesenchymal cells, chondrocytes, osteoblasts, dermal fibro-blasts, adipocytes, myocytes, odontoblasts, pulp cells). In line with this, the evolutionary role of the neural crest is outstanding without doubt, as those cells enabled the articulated jaws, teeth, complex vertebrate heart, advanced neural and hormonal regulation, efficient sensory modalities, efficient camouflage and much more. In 1983, Gans and Northcutt suggested the hypothesis of a “new head”, in which the neural crest played a major role in the acquisition of the head compartment as we know it in vertebrates [1].

In the early embryo, neural crest cells (NCCs) originate from the border of the neuroectoderm approximately at the time of neural tube closure. These cells undergo epithelial-to-mesenchymal transition (also known as delamination in this context) to exit the epithelial layer and temporarily acquire mesenchymal properties necessary for migration. On their way, NCCs follow specific navigational clues and become exposed to an array of factors directing their future fate.

While migrating, many NCCs associate with the outgrowing nerves and turn into Schwann cell precursors (SCPs) and boundary cap cells (treated here as a subtype of SCPs) [2]. Before the onset of myelination of the nerves, SCPs maintain multipotency similar to the neural crest and use nerves to navigate to distal locations in the growing embryo [3]. Thus, SCPs serve as a transitory state critical for the formation of late neural crest derivatives, including melanocytes [4], parasympathetic neurons [5,6], chromaffin cells [7,8], oxygen-sensing glomus cells [9], enteric neurons and glia [10,11] and posterior sympathetic neurons [8]. Furthermore, SCPs are capable of contributing minor quantities of cells to mesenchymal components of the teeth [12] and to the skeletal elements [13]. Thus, SCPs might be considered as a nerve-associated variation of the neural crest. Despite profound knowledge of signals and extrinsic properties affecting the fate of NCCs and SCPs, the question of fate choice has remained enigmatic and attracted a lot of attention throughout the last century.

**Fig. 1.**
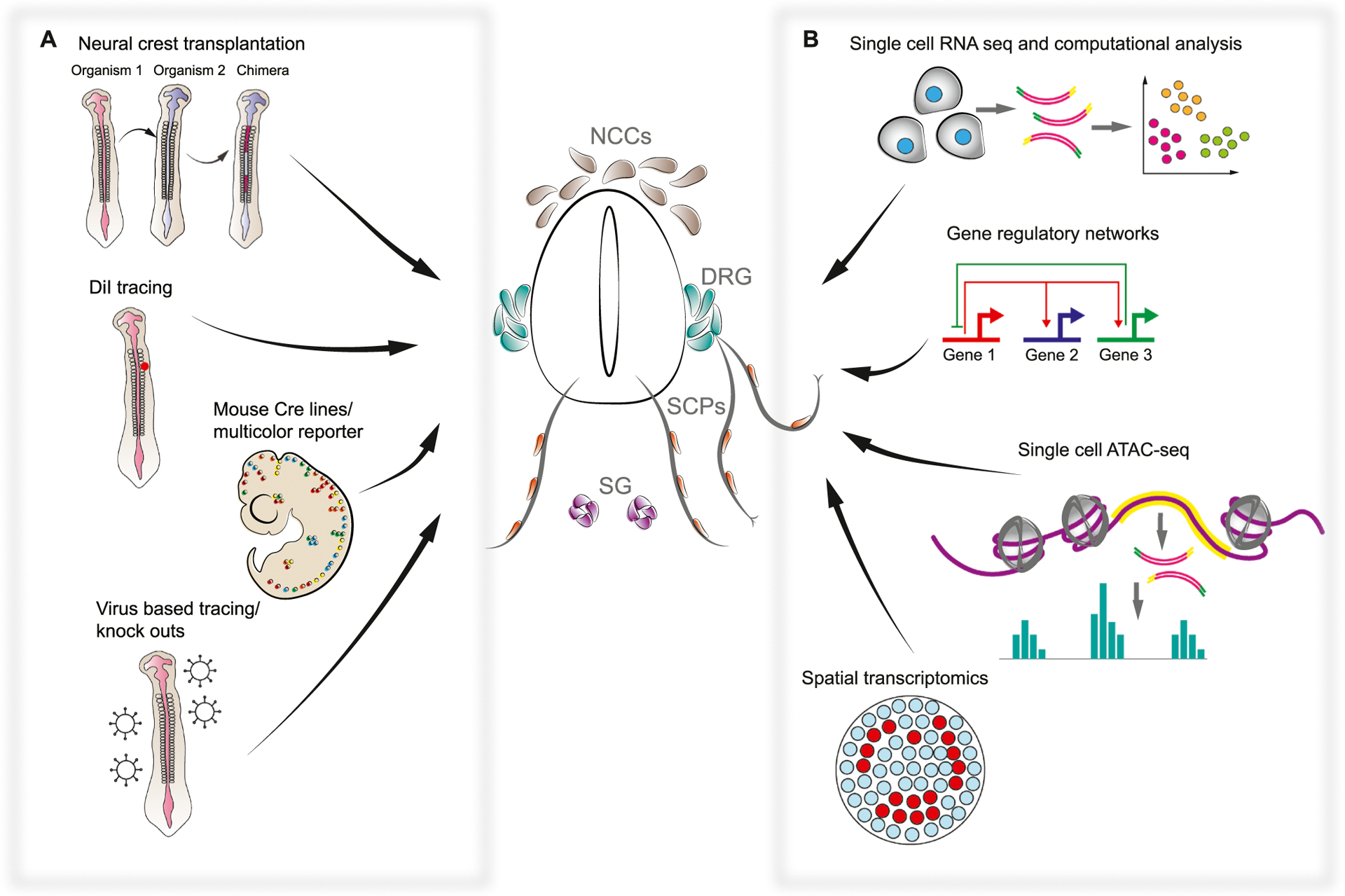
Neural crest potential and tissue contribution revealed by classical and new approaches. A. The suite of classical approaches to study the neural crest fate potential is presented by interspecies neural crest primordia transplantation done in multiple species; vital dye DiI tracing in chick embryos; transgenic mouse models with neural crest-specific Cre lines and multicolor reporter models; transgenic studies utilizing viral vectors for tracing and genetic functional analysis. These approaches allowed us to investigate what fates are generated by the NCCs and identified questions about neural crest clonality, multipotency, and heterogeneity. B. The suite of emerging genetic and transcriptomic approaches applied in the field of the neural crest studies is presented by single cell RNA sequencing with the following analysis of gene expression dynamics; investigation and construction of complex gene regulatory networks; single cell sequencing of open chromatin regions (ATAC-seq), which allows access to epigenetic gene regulatory control; and the recent advancement of spatial transcriptomics, which allows studying cells-to-cell interactions in a tissue context. These recent approaches allowed us to get closer to the understanding of how cell fate decisions are made by the individual NCCs and how the diversity of neural crest derived cell types is achieved. NCCs – neural crest cells, DRG – dorsal root ganglia, SCP – Schwann cell precursor, SG – sympathetic ganglion.

The pioneering experiments of experimental embryologists revealed the ability of the neural crest to give rise to different cell types under influence of different cellular environments (Fig. 1). This knowledge was achieved as a result of sophisticated grafting of region-specific neural crest to different parts of the body, across developmental stages, and between related species with different morphology. Indeed, the transplantation and classical embryological approaches dominated this field for some time, with researchers grafting various portions of neural crest onto different axial levels or species to understand their developmental potential. Using transplantation of neural crest among closely related amphibians, the gill arch skeleton and pigmentation were determined to depend on intrinsic properties of NCCs rather than their migratory environment [14,15].

In the 1970’s Nicole le Douarin brought cellular resolution to transplant studies by developing chick-quail chimera technology in which donor quail cells, based on antibody staining for their condensed heterochromatin, could be distinguished from chick host cells [16]. Using these chimeras, analogous stages and anatomical regions could be used to perform systematic fate-mapping of grafts at many axial levels, exposing tissue derivatives unique to neural crest subpopulations (Fig. 1A). For instance, vagal and sacral neural crest streams were found to be responsible for a large portion of the enteric nervous system [17]. In general, trunk NCCs were found to contribute to the dorsal root and autonomic ganglia, glial cells, and melanocytes, and seemed not to produce mesenchymal derivatives [18]. On the other hand, it was found that cranial neural crest, in addition to giving rise to cranial ganglia, are responsible for a large portion of mesenchymal derivatives in the face, including skeletal tissues, connective tissues, and arteries [19]. These studies discovered that different derivatives emerge from different axial levels, but the reason for a “positional fate code” remained mysterious.

Next, to characterize the heterogeneity among neural crest subpopulations, quail-chick chimeras in combination with diverse grafting conditions were used to reveal the developmental competence of NCCs. These experiments showed that at least some cells, within some neural crest subpopulations, have the potential to respond to a different environment and mimic a different crest subpopulation. For instance, the competence for pro-skeletal differentiation of jaw structures, which typically manifests in early-migrating cranial neural crest, is retained by later-migrating streams of cranial neural crest that don’t normally produce jaw structures [20]. It was also noted that grafting presumptive first arch crest onto presumptive second/third arch crest resulted in ectopic beaks, arguing that axial patterning can override the post-migratory environment [21]. Cranial crest also formed skeletal nodules when grafted into the trunk, whereas trunk crest could not even when grafted to cranial locations, suggesting cell-intrinsic differences in the developmental potential of crest along the AP axis [18]. With such approaches it became clear that distinct parts of the neural crest population do not have the same potential to generate all neural crest fates.

Multipotency of neural crest, revealed by transplant and chimeric studies, could be attributed to entire grafted cell populations, but caveats concerning genetic purity and lack of clonal resolution prevented strong conclusions about the multipotency of individual NCCs. Since then, diverse experimental setups were developed for lineage tracing. By injecting fluorescent dyes such as DiI and dextran (LRD) to migrating NCCs in chicken embryos [22,23], it was found that some individual migrating NCCs retain the ability to give rise to multiple fates in vivo (Fig. 1A). This approach also confirmed a previously hypothesized time-dependent complexity for neural crest fate selection, with earlier waves of crest contributing to both ventral and dorsal structures, whereas later waves of migration are dorsally restricted. Even though the dye-labeling approach afforded unambiguous clonal analysis for the first time, the throughput of one clone per embryo was too low to make general claims about NCCs. Further, dividing cells dilute vital dyes, complicating interpretation and precluding long-term dye tracing. The next big improvement of the in vivo clonal tracing of neural crest was transgenic mouse lines (in vitro approaches will not be described in this review but are reviewed in [24]).

**Fig. 2.**
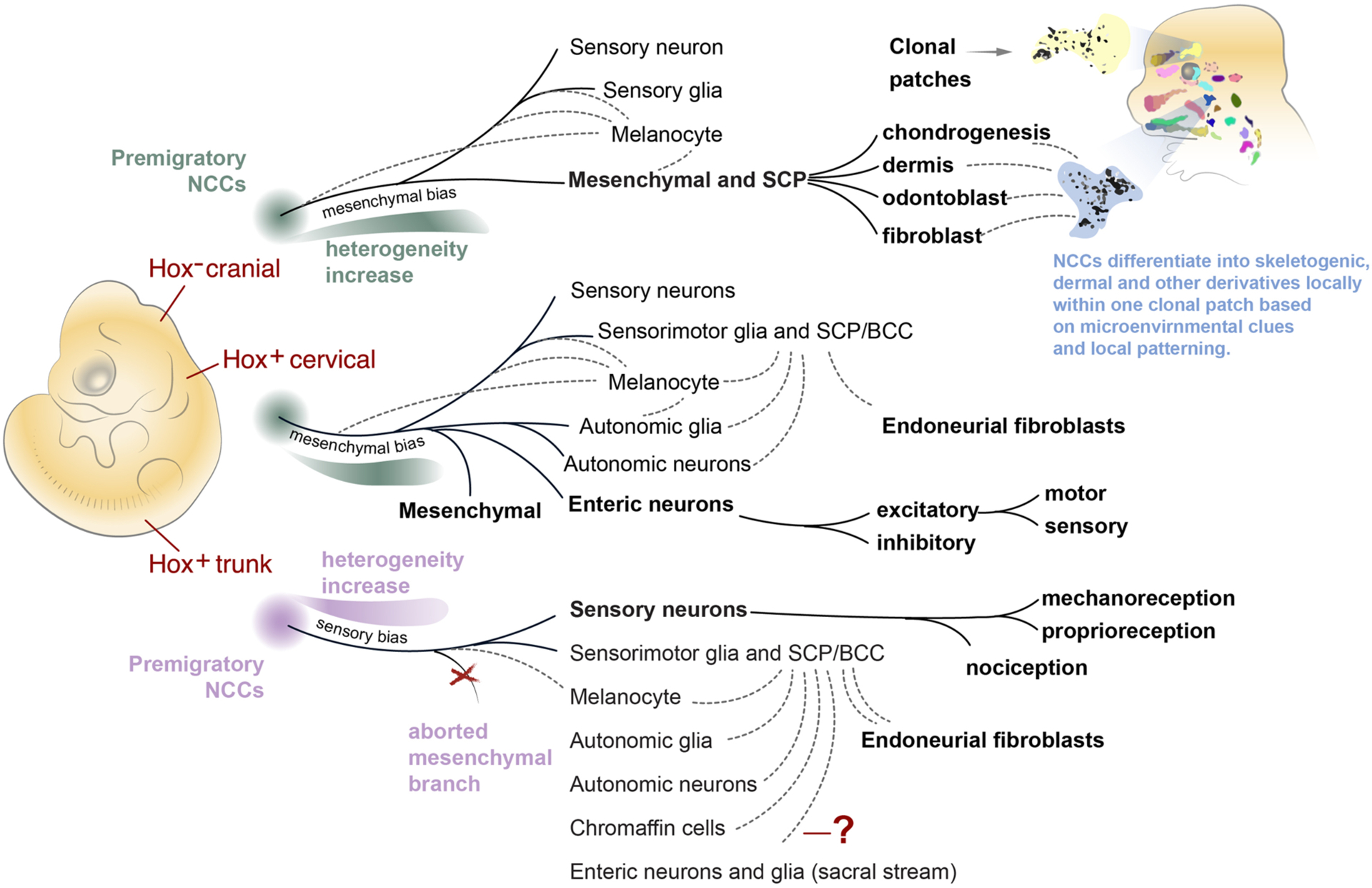
Single cell transcriptomics revealed new aspects of the neural crest lineage separation and fate landscape. NCCs give rise to different suites of cell types at different axial levels, but the sequence of decisions required to produce each fate might be fundamentally similar. Here, fate landscapes of three distinct axial levels in the embryo (cranial, vagal, thoracic) are illustrated with their predicted structure based on recent single cell studies. Solid lines represent major fates (also shown in bold) generated by the different levels. Dotted lines represent fate convergences, where multiple branches are predicted to contribute at different times to the glial, melanocyte, mesenchymal, autonomic, and other fates via intermediate cell progenitors such as SCPs and boundary cap cells. **(Top)** Cranial Hox negative NCCs are predominantly biased towards mesenchymal fates. Note that a very early split between mesenchymal and neurogenic fates is predicted because a smooth transcriptional transition between these fates has not yet been definitively shown in cranial NCCs populations. Cranial crest-derived ectomesenchyme is oligopotent in the face and differentiates as needed into various skeletogenic or connective tissue subtypes locally, while keeping recognizable physical boundaries of clonal patches. **(Middle)** The early cell fate decisions of the cervical/vagal Hox^+^ crest are predicted based on the deep single cell sequencing of NCCs at E9.5. This structure (sensory split first, followed by mesenchyme/autonomic split) represents the prediction that cervical Hox^+^ and trunk NCCs navigate the same series of stereotypical decisions on the transcriptional landscape. Further transcriptional profiling of the ENS branch, which is largely derived from the vagal crest, revealed several bifurcations between ENS neuron subtypes. **(Bottom)** The trunk neural crest is biased towards sensory neuronal differentiation rather than mesenchymal differentiation, but the decision-making co-activation step between conflicting mesenchymal and autonomic programs can still be observed in trunk crest cells. Thus, the branching point towards mesenchyme might be aborted by overwhelming fate biases caused by the signaling environment in the trunk. The next steps of the trunk sensory neuron subtype specification were revealed at the single cell level as a sequence of downstream fate decisions resulting in a plethora of sensory neuronal subtypes. NCCs – neural crest cells, SCP – Schwann cell precursor, BCC-boundary cap cell.

Cre-LoxP technology revolutionized cell lineage tracing by offering heritable and cell-type specific fluorescent markers (Fig. 1A). Many Cre and inducible CreERT2 drivers in mouse have been used to study neural crest progeny, but the most common are *Wnt1-Cre* (labeling dorsal neural tube), *Sox10-Cre*, and *Plp1-Cre* lines (labelling delaminating and migratory crest) [25–28]. In combination with the Confetti transgene, which stochastically expresses a combination of four fluorescent proteins in recombined cells, it is possible to analyze the multipotency of the neural crest at the level of individual clones. This was done in mouse at trunk [29] and cranial [30] axial levels to discover that clones of individual migrating trunk NCCs could give rise to sensory, glial, autonomic, and melanocytic lineages, supporting the notion of NCCs as *bona fide* stem cells. Interestingly, the ectomesenchymal clones observed in Kaucka et al. were spatially restricted, but not fate-restricted within a “clonal envelope” (see Fig. 2) suggesting that further choice of a differentiation trajectory in cranial mesenchyme depends on extrinsic signals and local patterning codes. For instance, skeletogenic and non-skeletogenic fates were systematically observed within the borders of a single clonal cluster in the developing face [30].

Avian model systems employed gene electroporation and replication-incompetent retroviral infection for lineage tracing and perturbations (Fig. 1A) [31–34]. These approaches permit diverse genetic manipulations, such as overexpression of fluorescent proteins that localize to specific different cell organelles [32], or of constitutively active or dominant negative gene products for functional assays [33], allowing researchers to identify many key molecular regulators of neural crest formation. In addition, the temporal flexibility of gene electroporation was used to reveal a dynamic spatiotemporal fate map at the level of the neural tube, suggesting an alternate model of early fate restrictions [34]. Krispin et al. meticulously explained how avian neural crest lineages were produced in ventral to dorsal order (first sympathetic, ventral root, sensory, finally melanocytes), from different waves of the neural crest that migrate at different times. Contributions of the crest could be predicted from their emigration timing, which depends on their dorsoventral position in the neural tube, and mostly fate-restricted clones were observed. As a result, cells most dorsally positioned in the neural tube would migrate first, and tended to contribute to the autonomic nervous system, whereas the cells in the more ventral cell layer migrated later, and largely contributed to the sensory and other fates [34]. Neurogenic crest rerouted to the dorsolateral (melanocytic) pathway by *Ednrb2* misexpression maintained neuronal characteristics, raising the possibility of fate-restricted pre-migratory neural crest in avian trunks.

The controversy about the degree of neural crest multipotency resulting from different experimental results [29,34,35] can be linked to the diversity of model organisms, axial levels, and methodological approaches used. Lineage tracing using combinations of fluorescent proteins activated in response to Cre-mediated recombination, such as Confetti technology, have been helpful to follow the descendants of specific cell types or gene expression domains, and investigate their clonal composition. For cell populations that remain relatively stationary, these methods are perfect for unambiguously defining clonal relationships [30,36]. However, these techniques have limitations tracing migratory stem cells. For instance, NCCs and their descendants are able to migrate along the anterior-posterior axis up to a distance of several somites, complicating interpretation of clonality on sections [37]. Reducing the labeling by administering extremely low doses of tamoxifen runs into natural limitations with the stochasticity of Cre-mediated recombination and suffers from the same low-throughput as dye-tracing. As results obtained by multiple studies agree that some trunk NCCs are multipotent, while other studies find evidence of early fate restrictions, it is likely that both mechanisms coexist in different neural crest subpopulations, but a definitive experimental test of such a hybrid model remained technologically inaccessible, until now.

## 2. Progress of single cell research in relation to the neural crest

The theory of epigenetic landscape presented by Conrad Waddington (1957) dictates that stem cells irreversibly decrease their developmental potential during development to produce distinct cell types [38]. Nowadays, our understanding of the Waddingtonian landscape has been transformed by a recent trend of studies employing single cell biology. The single cell field has now collected many sophisticated atlases of transcriptomic profiles across multiple species, gaining us insight into general developmental mechanisms. Such atlases describe heterogeneity of cell types across development in mouse [39–44], human [45,46], zebrafish [47–49], frog [50], and sea squirt [51]. Many of these studies did not focus strictly on neural crest development, but some crest transcriptional trajectories were shown and deserve a deeper analysis. An integrated embedding of the available neural crest lineages from these datasets would help uncover the transcriptional mechanisms of cell fate decisions at the neural plate border driving crest formation, and might reveal early-stemming fate biases that persist later in development.

Joint analysis of datasets from distantly related species, acquired using different sequencing platforms, remains a massive challenge for future evolutionary studies that wish to take advantage of these data, although some steps are already being taken to minimize platform-dependent batch effects [52]. Along these lines, comparative analysis of frog and fish development made evident various evolutionary changes to the fate landscape of neural crest [50]. One small example of this: the pigment cells, xanthophores, seem to emerge directly from an early population of NCCs in zebrafish, but only after multiple apparent intermediate progenitor states in frogs. Although it is difficult to draw meaningful evolutionary conclusions with so few species being compared, once atlases become available for many closely related organisms, entire fate landscapes might be used to uncover the mechanisms underlying the rapid evolvability of NCCs.

When applied to the neural crest, single cell studies made great strides towards cracking a few historically difficult questions about the structure of cell heterogeneity in the crest population and corresponding hierarchy of cell fate decisions [39,53–55] (Fig. 1B). These studies demonstrated that knowledge of early NCCs heterogeneity helps to identify early fate biases, thus putting our understanding of dynamic NCCs fate regulation into an appropriate context.

The recent works from Marianne Bronner and Tatjana Sauka-Spengler groups revealed the heterogeneity within the pre-migratory NCCs at single cell resolution in the chick model system (Fig. 1B) [53,55]. Williams and coauthors analyzed single cell chromatin and transcriptional profiles of avian cranial NCCs and found several populations, including some with pro-neuronal characteristics, among the pre-migratory NCCs still embedded into the dorsal neural tube [53]. Their single cell ATAC sequencing data suggests that NCCs prepare transcriptional machinery by opening certain regions of chromatin and expressing some genes to be used later during fate specification [53]. Moreover, the multi-modal approach of Williams et al. uncovered unique super-enhancers and gene regulatory elements that, together with core neural crest transcriptional factors, are essential for neural and non-neural fate decisions [53]. Indeed, single cell resolution allowed for the first time to identify specific enhancer regions of the neural crest specifier gene *Snai2* which are active only in the NCCs [53]. It also became clear that *Sox10*, being one of the central genes in establishing neural crest identity, is regulated by redundant super-enhancers to ensure robust expression [53]. Notably, *Sox10* belongs to a family of ancient SoxE transcription factors, and for *Sox10*, the determined crest-specific enhancer activity is preserved in jawless and jawed vertebrates, hinting at evolutionary conservation of key factors driving neural crest identity [56].

In another study with single cell resolution, Lignell et al. added spatial context to gene expression in individual NCCs at the pre-migratory stage (Fig. 1B). By combining fluorescent probes for 35 genes including general markers of pluripotency and specific markers of the crest, the authors revealed that early pre-migratory chick NCCs could be split into several distinct populations organized by their position in the dorsal neural tube. Principal genes contributing to heterogeneity among these populations encoded a range of functionally different molecules such as *Krt19, Col2a1, MycN* [55].

Finally, in a study by Soldatov et al., comparative analysis of murine cranial and trunk neural crest by single cell RNA-seq and spatial transcriptomics allowed reconstruction of neural crest lineage progression and cell fate decisions in a spatiotemporal context (Fig. 1B) [39]. The authors proposed a new model of neural crest fate diversification based on sequential binary fate splits, which we discuss below in more detail. Overall, by building and validating mathematical models based on the repertoire of currently available neural crest single cell data, we have a chance to uncover the modes of cell fate decisions and to understand the mechanism of neural crest lineage induction and diversification.

Although the methodology of single cell applications is not new anymore, there remain notable caveats. We do not aim at detailing the importance of reducing cell isolation time, which can introduce a stress signature into transcriptomes, or other technical issues including tricky computational analysis, which has been reviewed recently [57]. A greater danger comes from a potential lack of temporal resolution, which can lead to developmental processes appearing compressed or missing entirely. In the worst case, the temporal bridges between populations of progenitors and definitive cell types might not appear or emerge in a highly unstable manner. The depth of sequencing is another aspect, as low coverage will hide important regulatory molecules such as receptors, which are important for cell-cell interaction analysis [57]. Finally, we would like to bring up the key importance of the spatial aspect of single cell data, which is lost in classical droplet- or plate-based methods. Indeed, knowledge of tissue architecture is essential to investigate plausible cell signaling interactions as they relate to cell fate selection. As an example, when NCCs migrate next to the developing dermomyotome, they express Notch and other putative signals, predis-posing mesoderm to a subset of future fates [58]. Many developmental patterning mechanisms are still enigmatic, and might be discovered when researchers apply a bundle of spatial transcriptomics alongside other recent high-throughput approaches.

## 3. Single cell analysis reveals bifurcations of the neural crest fates and their peculiarities

One major unanswered question is whether different neural crest populations utilize unique Waddingtonian fate landscapes, or manifest differential biases while navigating a single integrated fate landscape. Soldatov et al. deeply sequenced transcriptomes of hundreds of NCCs to reconstruct the principal transcriptional trajectories throughout the early stages of neural crest development [39] in the form of a fate landscape, summarized in Fig. 2 for cranial and trunk populations. This fate landscape has been elaborated further in subsequent studies focusing specifically on sensory [59] and enteric [60] derivatives. According to this dataset, as soon as trunk NCCs undergo delamination from the dorsal neural tube and begin migrating, fate biasing is evident at the transcriptional level. Analyzing the topology of the landscape, the primary fork splits sensory (*Neurog2, Pou4f1, Neurog1, NeuroD4, NeuroD1)* from other lineages [39]. If sensory fate is not chosen, a second major fork clearly splits autonomic from mesenchymal lineages. Importantly, transcriptional evidence of the autonomic/mesenchymal fork can also be observed in the posterior trunk, even though no mesenchymal derivatives are generated there.

Because some cell types can be produced by multiple distinct progenitor populations, transcriptional “fate convergences” can also be observed in single cell data. This complicates traditional representations of cell lineages, which are typically based on bifurcating tree structures. This challenge was recently faced by Adameyko lab, when they tried to define and nest the melanocyte branch into the dataset of E8.5–E9.5 mouse NCCs. The multiple origin of melanocytes, including migratory NCCs in different phases and nerve-associated SCPs, precluded the formation of a single melanocyte branch from a well-defined root. Instead, melanoblasts (*Tyr*, *MITF*) emerged sporadically from all other branches within the embedding. Scarcity of a melanocyte gene expression signature might be expected at such early developmental stages, when melanocyte differentiation is by far incomplete and temporally heterogeneous across locations. Similarly, multiple trajectories originating from both major branches (sensory and autonomic/mesenchymal) and converging towards a glial cell state (*Sox10, Plp1, P0*) was also manifested by RNA velocity analysis [61]. These observations subtly encourage the speculation that glial or melanocyte cell types may represent a default terminal fate of the NCCs.

Unlike delaminating trunk NCCs, which show pro-sensory neurogenic bias, a strong mesenchymal bias (based on *Twist2* and other genes) dominates the cranial neural crest [39]. Anterior-posterior patterning might drive such early biases, which are already present during delamination, especially since positional gene expression signatures are retained in the corresponding migrating neural crest streams [39]. Notably, these region-specific programs include a significant number of genes beyond the Hox code [39]. The identification of pre-migration fate biases by recent single cell studies [39,53,55] is supported by lineage tracing studies generated by Chaya Kalcheim lab, where the pre-migratory NCCs located in different layers within the dorsal neural tube structure contributed to predominantly different parts of the neural crest lineage. As a conclusion, the dorsal neural tube environment emerges as a highly dynamic area [62], where the individual cells of the neural crest lineage change positions, and possibly transcriptional states, leading to pre-migratory micro-heterogeneity. In other words, anteroposterior patterning of the dorsal neural tube might drive the early biases detected at the delamination stage in the cranial and trunk neural crest populations, which in turn, might explain the differences in the resulting suite of cell types derived from each level.

Enteric neurons and glial cells are generated in multiple waves, from vagal NCCs populating the gut primordia starting from E9.5 in mouse (E3.5 in chick) and from sacral NCCs from 12.5 in mouse (E4 in chick) [17,63–66]. A third wave of enteric neurogenesis, derived from SCPs, constitutes up to 20% of cells [11]. Deep sequencing of single neurons from myenteric plexus isolated from a pan-neuronal *Baf53b-Cre* mouse line allowed classification of 12 enteric neuronal subtypes [60]. RNA velocity analysis revealed that the diversity of neurons in myenteric plexus is achieved by a major binary fate split between prototypic excitatory (*Calb2* ^+^) and inhibitory (*Nos1* ^+^*Npy*^+^) motor neurons identities, and confirmed a multilineage priming-based mechanism of fate segregation. Unexpectedly, further specification of neuronal subtypes occurs within those major fates by postmitotic differentiation. Some neuronal types differentiate within the prototypic classes by activation of the markers of late differentiation. For other types, trans-differentiation is observed. For example, *(Gad2* ^+^ *Neurod6* ^+^*, Rprml*^+^) interneurons and (*Nxph*^+^ *Ntng1* ^+^ *Calb1* ^+^ *vGlut2* ^+^) intrinsic primary afferent neuron specification occurs through a downregulation of *Nos1* and upregulation of a cholinergic program (*Scl18a2*) within the inhibitory neuron prototype [60].

Faure et al. further characterized the sensory neuron lineage stemming from trunk neural crest using similar Cre driver lines and found additional forks – adding to the overall trunk neural crest fate landscape [59]. They found the first fate split occurring between neuronal (*Isl1* ^+^) and non-neuronal crest or glial fates (*Sox10* ^+^ and *Sox2* ^+^). During this fork, cycling cells in the progenitor population activate pro-neural transcriptional factor Neurog1 and Neurog2. Moreover, both Neurog1 and Neurog2 are simultaneously expressed in individual cells of the first and second waves of sensory neuron specification. Because of technical limitations prior to the advent of single cell transcriptomics, these factors were previously considered exclusive for the first wave and the second wave of sensory neurogenesis [67]. Therefore, Neurogenin-dependent waves of sensory differentiation contribute to the new general model of NCC fate decisions where multilineage priming occurs prior to transcriptional bifurcation. The next major sensory fate split partitions the unmyelinated nociceptive lineage (*Runx1* ^+^*, Ntrk1* ^+^), followed by the separation of myelinated proprioceptive (*Ntrk3* ^+^*,Runx3* ^+^) and finally mechanoreceptive (*Ret*^+^*, Ntrk2* ^+^) lineages with further specification of mechanoreceptive cells. As a proof of principle that transcriptional factor competition drives binary cell fate decisions, functional tests confirmed that *Runx3* ablation in mouse leads to overrepresentation of mechanosensory fates [59].

**Fig. 3.**
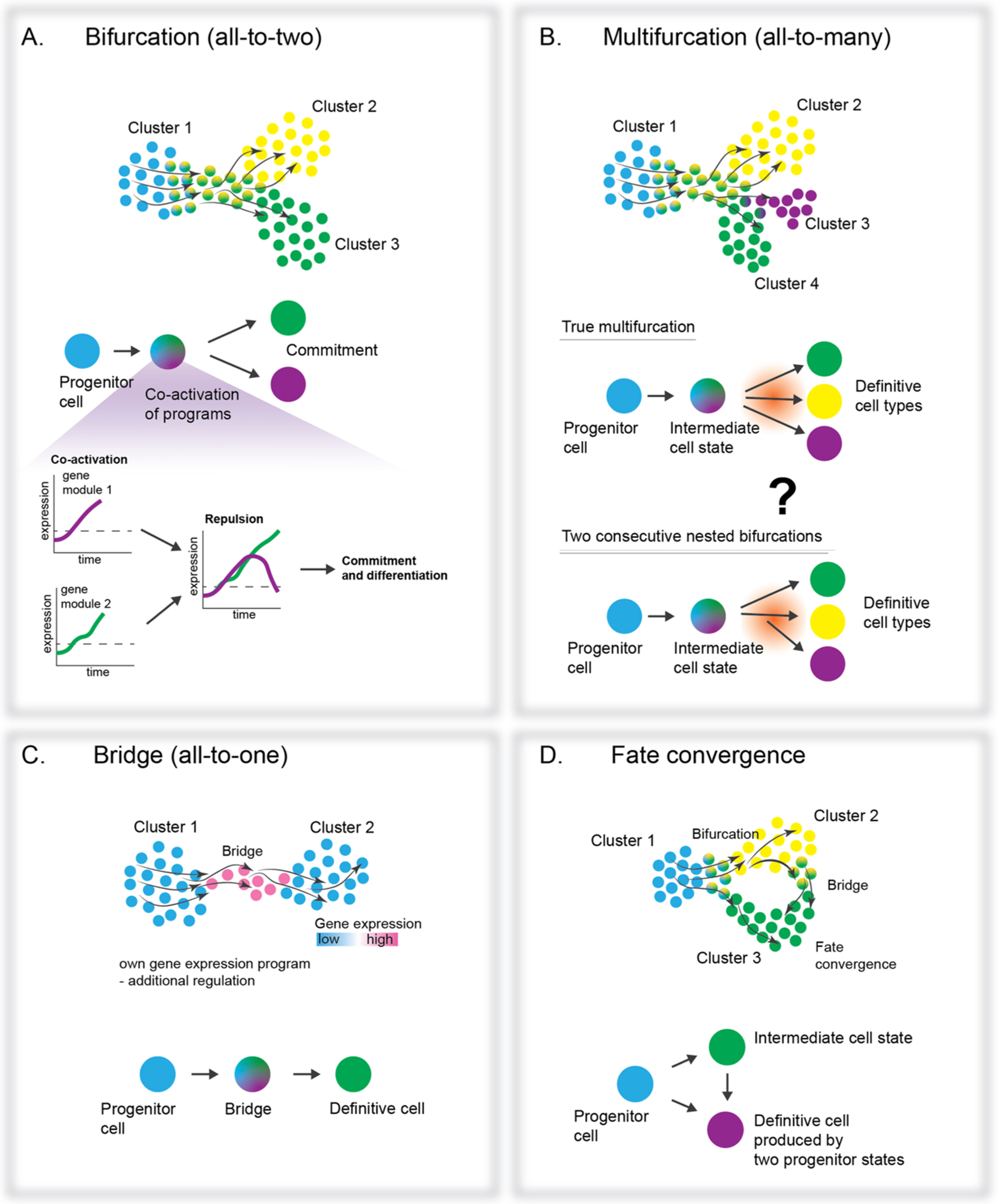
The diversity of transitions revealed on transcriptional landscapes. Single cell transcriptomics of cell populations, coupled with RNA velocity analysis, visualizes transcriptional dynamics as vector fields within a projected 2D transcriptional space, making it possible to assess features of the NCC fate decisions and resulting transitions. At least three motifs emerged as basic units representing transitions on the transcriptional landscape: bifurcation, multifurcation (still missing a reliable proof of existence), bridge, and fate convergence. A. Bifurcation occurs when a population of precursor cells decides between two distinct fates. The process features co-activation of fate-specific programs within each cell causing the development of micro-heterogeneity, and cross-repulsion of the competing programs. Eventually, one program wins, being immediately followed by fate segregation at the transcriptional level. B. Multifurcation is similar to a bifurcation with the number of potential fates being more than two. However, some uncertainty remains whether currently predicted multifurcations are in fact closely nested bifurcations (see question mark). C. Analysis of transient cell states have identified and transcriptionally characterized the intermediate cell states (bridges) that mediate the transition between progenitors and definitive cell populations. **D.** NCCs (and other progenitors in the body) often take different transcriptional routes to reach the same endpoint (definitive fate) resulting in convergence. These routes may feature differential susceptibility to extrinsic signaling events, which offers an additional regulatory level on clonal diversification.

It appears that a remarkably similar logic drives cell fate decisions in the entire neural crest lineage, from early progenitors escaping the neural tube, to the final differentiation of the terminal cell types within sensory and enteric nervous system branches. These major processes (illustrated in Fig. 3) include fate bifurcations (and possible multifurcations), fate transitions (via intermediate bridge populations), and fate convergences. These well-defined, transient cell states may represent generalized transcriptional mechanisms of cell fate choice, and are described in detail in the next section.

## 4. Micro- and macro-heterogeneity of the neural crest in a context of a 3-step fate selection mechanism

Previously, Sui Huang and his team proposed a definition for cell fate commitment as “a critical state transition or tipping point at which complex systems undergo a sudden qualitative shift” [68]. Before such a tipping point can be reached, progenitor cells make a decision, which may depend on each cell’s accumulated lineage history, integration of the extracellular signaling environment, and epigenetic status [69]. Single cell transcriptomics captures fine changes in transcriptional states, some of which might reflect decision-making processes. Although major transcriptional changes are often thought to be associated with fate choice events, those might rather reflect post-commitment cell differentiation, whereas slight changes involving only a few genes might be truly responsible for symmetry breaking. New methods for tracking such fine events along developmental timing can identify subtle changes in correlated and non-correlated patterns of expressed genes [39,68,70].

A fate transition through a binary fate split (bifurcation) is usually observed when a seemingly homogeneous population of precursors splits into two cell types with different gene expression programs (Fig. 3A). Such events direct early NCC fate specification, as the activation of all possible fates does not happen simultaneously, instead proceeding through a series of consecutive forks [39]. The existence of multifurcations shall not be excluded, as it was recently reported during mammalian gastrulation [71]. However, definite proof of multifurcations remains a challenge because without sufficient resolution, closely nested bifurcations can always be misinterpreted as multifurcations (Fig. 3B). So far, multifurcations were not directly observed in the neural crest lineage, which seems to instead progress via fate bifurcations, linear fate transitions, and fate convergences.

Soldatov et al. reported a 3-step mechanism for fate bifurcations in mouse NCCs, including early co-activation of gene expression modules corresponding to two conflicting fates, followed by mutual repulsion of these modules [39]. During co-activation and repulsion phases, cells coordinately express emerging genetic modules to a different degree, resulting in increased transcriptional heterogeneity. Eventually, one module takes over in each cell to manifest specific fate commitments (Fig. 3A) [39]. Following the bifurcation, transcriptional profiles strongly diverge between cell populations committed to different fates. In line with these observations, previous research revealed increases in cell heterogeneity and coordinated gene expression before the bifurcation [68]. For instance, differentiating induced pluripotent stem cells exhibited coordinated emergence of gene regulatory networks related to future stable states, which could be interpreted as attractors on the Waddingtonian landscape [68]. Furthermore, Bargaje et al. identified biasing factors acting before the bifurcation point and applied this knowledge to predict the proportions of resulting cell types in a variety of experimental conditions [68]. Therefore, the quantitative analysis of compositional effects in differentially biased progenitor populations can predict fate balance, which is a cornerstone for improving *in vitro* cell differentiation protocols.

Although Bargaje et al. and Soldatov et al. generally used similar statistical approaches, Soldatov and coauthors partitioned correlations of expressed genes into the modules related to the resulting fates, which provided the measures of repulsion and attraction of the emerging fate-specific programs in the heterogeneous population of cells before the bifurcation [39,68]. Of note, the identified molecules and modules temporally associated with cell fate decisions might turn out being rather “passengers”, not the “drivers” of fate selection. It takes an experimental effort to identify expressed genes which functionally contribute to decision-making, while separating them from the genes that simply demarcate the decision-making phases of lineage development. Although models interpreting single cell data are prevalent in the literature, functional experimental validations are necessary to determine the roles of identified molecules. Nonetheless, statistical predictions facilitate this process by feeding researchers with fruitful educated guesses, and the observation of opposing genetic programs (albeit possibly dominated by “passenger” genes) represents a break-through in understanding cell decision-making.

Next, Soldatov et al. found that some cells in the pre-bifurcation state coordinately express one fate-specific gene expression module, but not the other. However, the majority of the crest cells revealed the co-existence of both opposing modules being assembled to various degrees [39], which can be interpreted as a multilineage priming of a varying strength (Fig. 3) [72]. This notion is supported by recent studies in frog and zebrafish which suggest that multilineage priming prior to bifurcations might be a universal cell mechanism for executing fate decisions during development [50]. Taken together, the observed multilineage priming suggests NCCs might respond to micro-environmental signals by tuning their individual fate biases during the gene module co-activation phase [39]. However, we cannot yet exclude the possibility that the investigated NCC populations might be intrinsically heterogeneous (e.g. epigenetic priming) prior to neural crest formation, which can also contribute to a compositional effect at pre-bifurcation states.

**Fig. 4.**
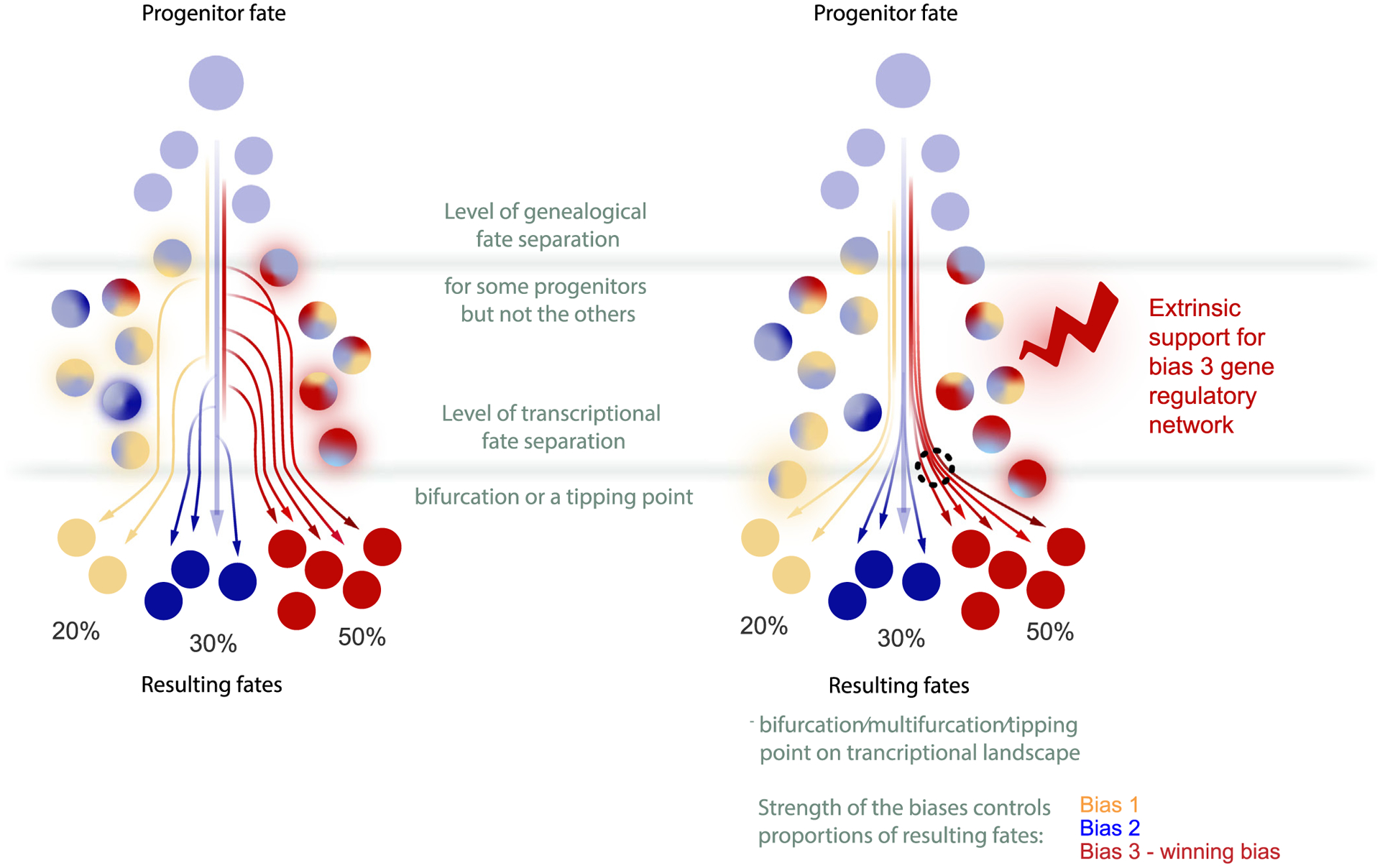
The conceptual difference between genealogical versus transcriptional fate separations. Schematic of the NCCs arranged on a transcriptional manifold undergoing multilineage priming, fate separation and differentiation. Unprimed progenitor cells are shown in light blue, and multicolored states indicate specific fate biases. Note that the progenitors may experience strong differential biasing along real time and space (approximated as a pseudotime on the transcriptional portrait). Some early pre-bifurcation biases might be strong enough to predetermine the outcome of fate choice (**left scheme**), even though the apparent transcriptional fate split for the entire heterogeneous cohort of differentially biased progenitors might only occur later (**right scheme**). Importantly, these initializing mechanisms of cell commitment may be hidden from transcriptomics-only approaches.

Both extrinsic and intrinsic modes of non-genetic heterogeneity create, within a given cell population, a distribution of fate biases (Fig. 4) [73]. For instance, Hannah Chang and coauthors showed that transcriptional variation in hematopoietic stem cells impacts the amount and the type of progeny. At the same time, transcriptional outliers are capable of reconstituting gene expression distributions of their mother population, suggesting the existence of attractor states with some degree of fuzziness [74]. Here, the term “attractor” is interpreted as “a stationary and stable network state into which a set of particular network states will eventually evolve” [73]. Functional identification of attractor states might be useful to attain the range and proportionality of cell types necessary to build tissues and organs from an initially homogenous population of multipotent progenitor cells. In addition, the stability of the path on the Waddingtonian landscape towards the attractor, i.e. the amount of tethering before the attractor state is reached, might define the plasticity of progenitor cells being exposed to combinations of fate-biasing signaling factors. Modeling this landscape might help determine if more natural differentiation approaches (as opposed to non-physiological or artificial cell reprogramming methods) can achieve completely pure populations of a single target cell type, or whether the stochasticity inherent to multilineage priming makes contaminating cell types practically unavoidable (for an example, please see [75]).

The sum of these ideas raises an important question of how the proportionality of cell types is achieved in the neural crest lineage (Fig. 4). Given that NCCs are, at least, significantly biased by known extrinsic signals [76], it is easy to imagine that signal transduction mechanisms can be tuned by evolution for adaptive thresholding prior to bifurcations to define the proportionality of resulting cell types. Therefore, such biasing molecules can be a perfect evolutionary substrate, as the changes in organ shape, size or function may result from simple changes in proportions of the otherwise conserved cell types instead of evolving new cell types or novel cellular functions. For instance, in the cranial neural crest, the fate splits in mesenchymal-biased neural crest precursors will eventually give rise to bones and cartilage of the face, and also to the neuro-glial populations of the head. Remarkably, the transient and mosaic expression of *Oct4/Nanog* in the cranial neural crest represents an important biasing factor, which favors specification and expansion of various ectomesenchymal cell fates. The neural crest-specific ablation of *Oct4* does not affect overall embryogenesis, except for the amount of ectomesenchymal cells giving rise to the frontonasal cell mass responsible for the formation of the face, without other detectable effects on other fates of the neural crest [54]. Thus, tuning the transient expression of pluripotency genes in the cranial neural crest might be a mechanism for controlling the size and shape of the face and snout.

The mystery of whether NCC subpopulations use differential biases to navigate a single fate landscape, or are presented with fundamentally unique decisions on separate landscapes, may be approached by identifying “muted” bifurcations where multilineage priming occurs but one fate is never expressed. Accordingly, Soldatov et al. revealed a fate conflict between mesenchymal and autonomic pro-neuronal branches in the posterior mouse trunk, where mesenchymal fates do not normally form [39]. This detection was based on the observation of competing mesenchymal and autonomic modules before the bifurcation point, although the mesenchymal bias appeared weaker as compared to the cervical or cranial regions. The existence of such conflicts suggests that the mesenchymal bias occurs in the trunk populations, but being weak and overpowered by neuro-glial bias, does not result in mesenchymal progeny. Despite the fact that mesenchymal fates are not born in that part of the mouse embryo, the *bona fide* bifurcation (although it is never realized practically) is still present and needs to be considered as such. One would expect that experimental strengthening of this weak mesenchymal bias could result in mesenchymal progeny in the trunk. This was exactly achieved by the introduction of cranial crest-specific *Twist2* into trunk neural crest, which is usually largely devoid of *Twist2* expression [39]. As predicted, overexpression of *Twist2* in the chick trunk neural crest resulted in a burst of mesenchymal fates. In more natural settings, a similar bias control might be responsible for the remarkable and unusual mesenchymal contribution from the trunk neural crest to the developing turtle shell [77]. Interestingly, such control resembles the situation observed in the development of the T-cell sub-lineage, as reported by Ellen Rothenberg group, where the progenitors of T-cells undergo multilineage priming for both myeloid/dendritic cell and innate/natural killer cell fates, which do not get realized in this context. In the end, progenitors commit to the T-cell fate due to the expression of *Bcl11b*, which desensitizes progenitors to the regulators of alternative fates [78].

Not only extrinsic biasing molecules or region-specific patterning might be responsible for macro and micro-heterogeneity of the early neural crest population. Temporal fluctuation is another source of intrinsic heterogeneity [73], though we still lack methodology to sample transcriptomic signatures in the same cells over time. Transcriptional oscillators (including the cell cycle), burst-based transcriptional dynamics, and gene expression noise, all might contribute to the primordial micro-heterogeneity in the neural crest lineage. At the same time, the heterogeneity detected by Soldatov et al. appeared to be largely caused by a structure of gradually assembling genetic modules corresponding to terminal fates, rather than oscillatory dynamics or noise [39].

Finally, pre-bifurcation heterogeneity in NCCs might affect the rate of differentiation, causing the decision-making of a cell population to appear rather like a dispersed area instead of a sharp point in pseudo-time. Indeed, in the cervical region of mouse embryos, cells undergoing fate bifurcation between mesenchymal and autonomic proneurogenic programs appear diffusely spread out in transcriptional space, compared to the primary sensory split. Notably, the corresponding neural crest streams generate the largest sympathetic ganglion in the body – the superior cervical ganglion, and, at the same time, contribute cells to the skeletogenic populations in the neck and cardiac neural crest. The fate conflict between the autonomic and mesenchymal fates pinnacles in co-activation of pro-mesenchymal *Prrx2* and pro-autonomic *Phox2b* master regulators [39]. Some cells co-expressing these and other factors manage to pass the bifurcation point without resolving the fate conflict and form a fuzzy cloud of cells with co-activated opposite programs, which instigates a question of what happens to such cells later on. Of course, some of them resolve the fate conflict in a delayed manner (as suggested by the lineage tracing data in [39]) or die. However, even if they resolve the fate conflict later in time, their epigenetic memory might not form normally, which might leave the opportunity for unwanted plasticity between autonomic and mesenchymal fates in the progeny of such delayed cells. Hypothetically, such plasticity might predispose these cells to neuroblastoma cancer transformation, which is known to contain plastic autonomic adrenergic and mesenchymal tumor cell types, which are capable of reconstituting each other in experimental settings [79]. Therefore, understanding pre-bifurcation dynamics might help to reveal the conditions leading to pre-malignancy and epigenetic origin of some pediatric tumors [80], for instance, in the case of some neuroblastoma subtypes with a low mutation load [81].

## 5. Bridges and linear transitions in the neural crest lineage development

Not all transitions proceed via bifurcation for progressing cell lineages. Many transitions proceed in a linear fashion and display transient intermediate states called “bridges” (Fig. 3C). Even in the case of linear transitions, progenitors need to choose whether to keep their current stable phenotype or to differentiate further. Thus, in agreement with Bargaje et al., this can also be considered as a type of a bifurcation in some way [68]. Treating stable states as attractors on the lineage development landscape, Bargaje et al. suggested decision-making begins with dissolution or destabilization of the previous attractor under the influence of signals impacting gene regulatory networks [68].

Fate transition via the “bridge” is observed when a continuum of cells is clustered connecting two cell types with or without their own unique gene expression programs. Therefore, such bridge states can correspond to a pure downregulation of the old state genes and an upregulation of the new state genes, showing a gradual mixture of two genetic programs along such transitions. Alternatively, the transitory state can reveal its own gene expression signature, rendering it closer to a true transitory cell type or less stable attractor with a specific gene expression program. One key example of such transition though the “bridge” in the neural crest lineage is represented by the differentiation of chromaffin neuroendocrine cells from SCPs [7]. Chromaffin cells start to emerge at E11.5, mainly from SCPs in mouse embryos. By this time, the free migration of the crest is finished at all axial levels, and all free NCCs have associated with nerves and become SCPs [2,3]. Deep sequencing of single cells from E12.5 and E13.5 murine sympathoadrenal anlagen revealed that SCPs transform into chromaffin cells through a narrow transient cell cluster called the “bridge” with the unique expression of marker genes including *Htr3a* and others [7]. The analysis of RNA velocity [61] and experimental lineage tracing confirmed the direction of differentiation from SCPs into chromaffin cells. Potentially, the “bridge” cell state might be important for controlling the numbers of chromaffin cells through a paracrine regulatory loop, as “bridge” cells express specific receptors complementary to local ligands produced by more differentiated cells. In the following studies of the developing neural crest and sympatho-adrenal system, other bridges and transitions were recently uncovered, including a bridge between SCPs and sympathetic neurons and another bridge between sympathoblasts and chromaffin cells [80]. Some of these bridges appeared unique only for human development, which might underpin the potential for tumor initiation only in humans and explain the origin of neuroblastoma in the adrenal gland area [80].

## 6. Fate convergence in the neural crest lineage

Fate convergence occurs when cell populations from different lineages, with different developmental competences, contribute to the same definitive or intermediate cell type [40,48,82–84] (Fig. 3D). There are numerous examples of fate convergence in nature, but as for the neural crest, a famous example was recently illustrated in a zebrafish single cell atlas, where mesodermal and NCCs converge smoothly onto an identical mesenchymal fate [48]. Such “fate convergence” loops were known to exist since long ago, given the historical efforts to determine which cranial bones were crest- versus mesoderm- derived [85]. Thus, it is possible to molecularly define each convergence point and track the emergence of similar fates through gene expression and epigenetic regulation. The resulting cluster of cells originating from different lineages will not show transcriptional heterogeneity, as that reflects gene expression tailored to actual cell function. However, future studies utilizing detailed epigenetic analysis and lineage recording technologies will provide insight to understand whether epigenetic memories of parent lineages exist in fate-converged cells.

In another example, the single cell transcriptomics analysis of murine neural crest did not result in identification of a melanocyte branch either in the trunk or in the cranial regions during the time of the neural crest formation and migration [39]. This strange result requires reconciliation with the classical model, where melanoblasts emerge as one of the late neural crest derivatives. The detailed analysis of the dataset published by Soldatov et al. reveals that a master regulator of melanocyte fate *Mitf* (in a combination with *Sox10*) becomes induced in various neural crest subpopulations, starting from the pre-migratory and delamination stages. Because the expression of melanocyte markers and master regulators of melanocyte fate does not achieve significant coordination and emerges in cells across multiple neural crest differentiation phases, a single and clear branch leading to the future melanocytes cannot be established during the phases of delamination and migration [39]. Furthermore, SCPs are known to generate a large proportion of melanocytes in the developing embryo [4,86–88]. Therefore, in mouse, melanocytes originate from multiple cell sources with diverging cell history over quite a long time, and, in the end, are convergently generated as a solid population. This process resembles the dynamics of fate splits during gastrulation, where the same fates could branch out multiple times from the evolving progenitor stem across a wider time window [71]. Thus, the same or similar bifurcations can be repeated multiple times from slightly varying ground states observed in different phases of development or spatial contexts. It remains an open question how different progenitor cells with distinct histories of spatiotemporal regulation manage to nonetheless consolidate the melanocyte fate.

Not only melanocytes are jointly generated from the nerve-associated SCPs and the migratory crest. Another example includes the sympathetic neurons [80]. The intra-adrenal [80] and some paraganglia-specific [8] sympathoblasts are generated from the local SCPs, whereas the sympathoblasts of the sympathetic chain are predominantly derived from the migratory NCCs. A similar fate convergence strategy is observed during the development of enteric neurons. Some of them are derived from the early neural crest streams, and some are derived later from the SCPs recruited from local (including extrinsic) innervation of the gut [10,11,89]. Overall, it seems that SCPs represent a population continually deviating from the neural crest, and, at the same time, retaining some of the neural crest multipotency and convergently giving rise to classical neural crest-derived cell types. Such a strategy might help to ensure the prolonged existence of a stem cell source, which is necessary during late and postnatal development, well beyond the transient time window of neural crest migration [3]. Further single cell studies should clarify how the recruitment of SCPs is initiated and how multipotency persists in these cells.

The characterization of numerous fate convergence in the neural crest lineage raises an important caveat regarding clonal history. Although reconstructed trees of transcriptional events, including sequential bifurcations, might accurately describe the changes of the internal states of cells progressing along the Waddingtonian landscape, those changes and the transcriptionally defined fate splits do not necessarily correspond to actual lineage as per clonal genealogy. Indeed, Tracer-seq [48] revealed that the graph topology of a time-resolved single cell dataset in zebrafish does not always reflect lineage histories of single clones when measured using CRISPR/Cas9-based clonal barcodes. Thus, on lineage-bearing transcriptional maps, clonal history and cell state have been effectively uncoupled, in part due to fate convergence in which the same cell state is produced via distinct progenitor pools. In another study from the Alon Klein lab, analysis of clonal segregation detected fate restrictions much earlier than predicted by the transcriptional landscape during hematopoietic lineage development *in vitro* and *in vivo* [84]. Overall, the early biasing of progenitors belonging to the same general cell type can drive fate restrictions which might be undetectable using transcriptional state maps alone.

## 7. Conceptual models of neural crest cell fate restrictions and multipotency

The preceding discussion hints that the question of whether stem cell populations utilize individual multipotency (when a cell *de facto* gives rise to all downstream fates) or collective multipotency (when a cell *de facto* gives rise only to some of the possible downstream fates) can be resolved only by blending single cell transcriptomics with clonal analysis. This thesis is perfectly applied to neural crest multipotency, having been analyzed and challenged experimentally in multiple species.

The discussions of how fate restrictions are implemented in the neural crest lineage have a long history, and can be briefly summarized as a bunch of partly overlapping opinions, mostly including the vision of NCCs undergoing fate restrictions based on local signals, but disagreeing on when and where the restrictions may take place. These modes of fate specification are outlined in a recent hypothesis paper by Robert Kelsh [90]. In brief, a “progressive fate restrictions” model suggests that NCCs, being initially multipotent, gradually lose their potential by committing to sub-branches of the fate landscape. This progressive model stipulates that the choice of differentiation route closes opportunities for alternative routes [90]. On the other hand, a “dynamic fate restrictions” model suggests NCCs remain multipotent and capable of generating any neural crest fates permitted by a given environment. According to the dynamic model, fate choice is less dependent on a cell’s history and more about committing to definitive cell types in response to post-migratory signaling environments.

**Fig. 5.**
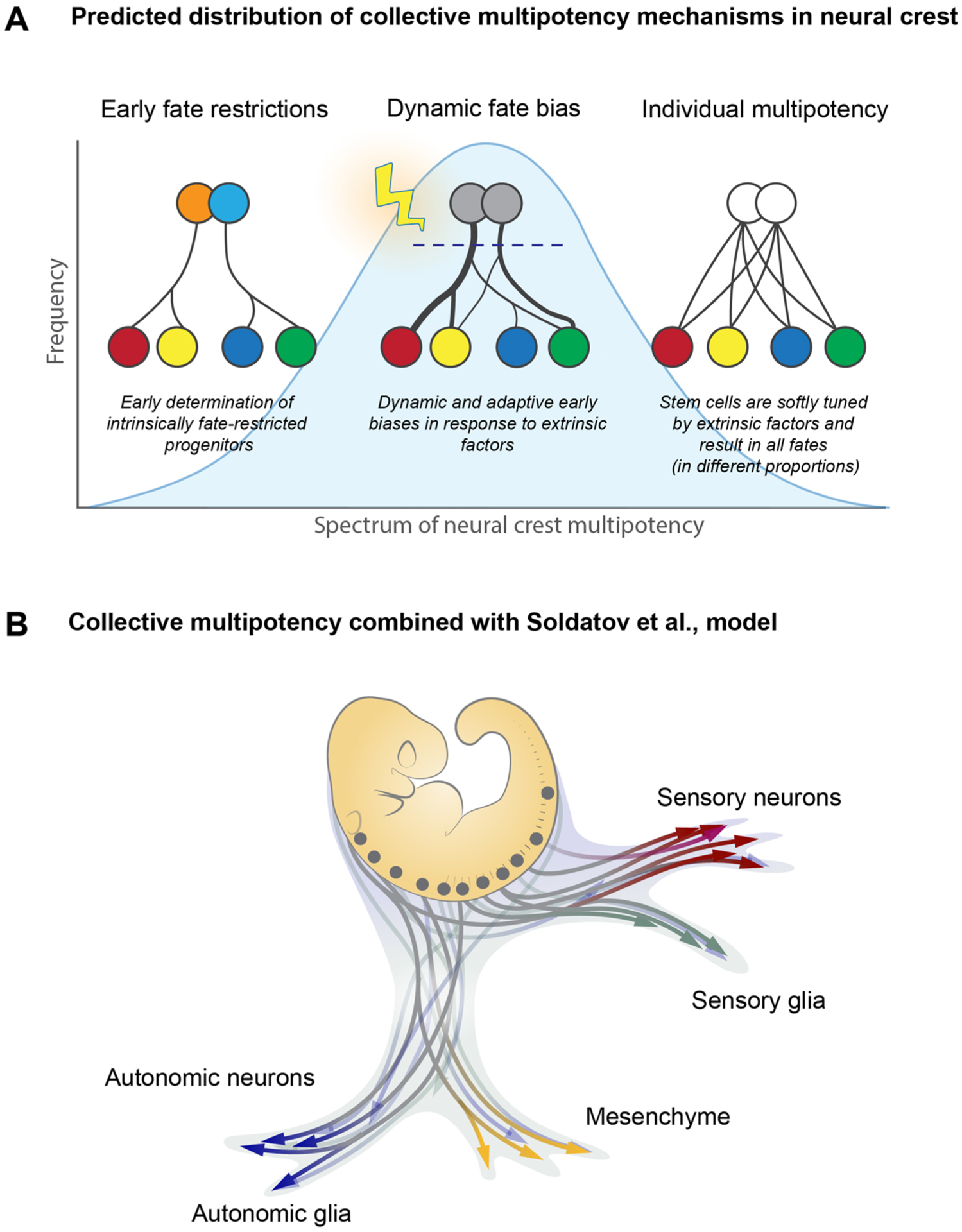
A hypothesis of the distributed fate potential across the entire population of the NCCs (collective multipotency hypothesis). A. Multiple competing models, such as “early fate restriction model”, “dynamic fate biases model” and “individual multipotency” model can be reconciled into one unifying framework of a multipotency distribution across the entire neural crest population. Within this concept, the formerly competing models are not mutually exclusive, but might be each utilized by the embryo with varying degrees of frequency. **(Left)** In the model describing early restrictions, the NCCs would be restricted to producing specific sublineages by the time of their formation in the dorsal neural tube and possibly even earlier. Thus, an “early restrictions” model imagines the neural crest as a mixed population of precursors (shown by differently colored circles) with distinct non-overlapping developmental potentials. At an extreme version of this model, some early restrictions may result in unipotent crest cells. **(Middle)** In the model describing dynamic fate biases, the individual NCCs become fate-biased and do not give rise to the entire spectrum of possible fates due to signaling factors present in the dorsal neural tube and later during cell delamination. The biases control the proportionality of fate allocation in the progeny of each NCC clone, and, as a result, individual NCCs will contribute only to a subspectrum of fates with varying degrees of overlap and contribution. **(Right)** In the model describing individual multipotency, the entire range of NCC fates is *de facto* produced by any of the individual NCCs. Thus, an individual multipotency model imagines the neural crest as a homogenous population of multipotent stem cells, each resulting in the full range of progeny. (B) An example of the entire neural crest populations navigating the fate landscape by utilizing a combination of modes of multipotency. Some arrows are highly branching, others are more restricted. At the same time, the sum of the individual NCCs results in collective multipotency covering all developmental needs.

Although individual multipotency was claimed for a number of model systems [29,91,92] this, in fact, might be true only for a portion of NCCs. Another part of the crest could be fate-restricted or undergo dynamic fate restrictions via early extrinsic biasing, never fully realizing the multipotency at the level of individual cells, as supported by the fate mapping studies from Chaya Kalcheim lab [34,93]. On a large scale, the neural crest might represent a spectrum of truly individually multipotent or pre-biased states (Fig. 5). Thus, the proportion of NCCs exhibiting collective versus individual multipotency could shift depending on the species, location, timing, and/or possible disturbances due to environmental and genomic perturbation.

Interestingly, Robert Kelsh and coauthors suggested a third model, which they called “cyclical fate restriction (CFR)” [90]. According to CFR, NCCs undergo dynamic and possibly cyclic transitions between fate-biased states, most likely under the influence of signaling molecules, until they commit to a specific subset of fates. During these cyclic or pseudo-cyclic transitions, the cells show co-presence of conflicting transcriptional programs, which attempt to drive cells to opposing fates [90]. This is reminiscent of the situation observed by Soldatov et al. in murine NCCs, where the opposing genetic programs bias and compete prior to the fate selection points along the neural crest differentiation trajectory. Thus, based on a sum of experimental observations of competing programs, it is natural to reconcile the CFR model with the mechanistic views on fate restriction developed by Adameyko and Kharchenko labs [39].

Ultimately, predictive understanding of neural crest multipotency requires a detailed study of the regulatory signaling mechanisms that produce spatiotemporally structured micro-heterogeneity in the dorsal neural tube, and how early-versus later-acting biasing factors drive specific clonal fate proportionality of NCCs at pre-migratory and delamination stages in the embryo. Future experiments with clonal labelling together with readouts of individual transcriptional states [84] will be necessary to distinguish the relative contributions of cell-autonomous, extrinsically-guided, and/or possibly cyclic modes of cell differentiation.

## 8. Conclusion

**Fig. 6.**
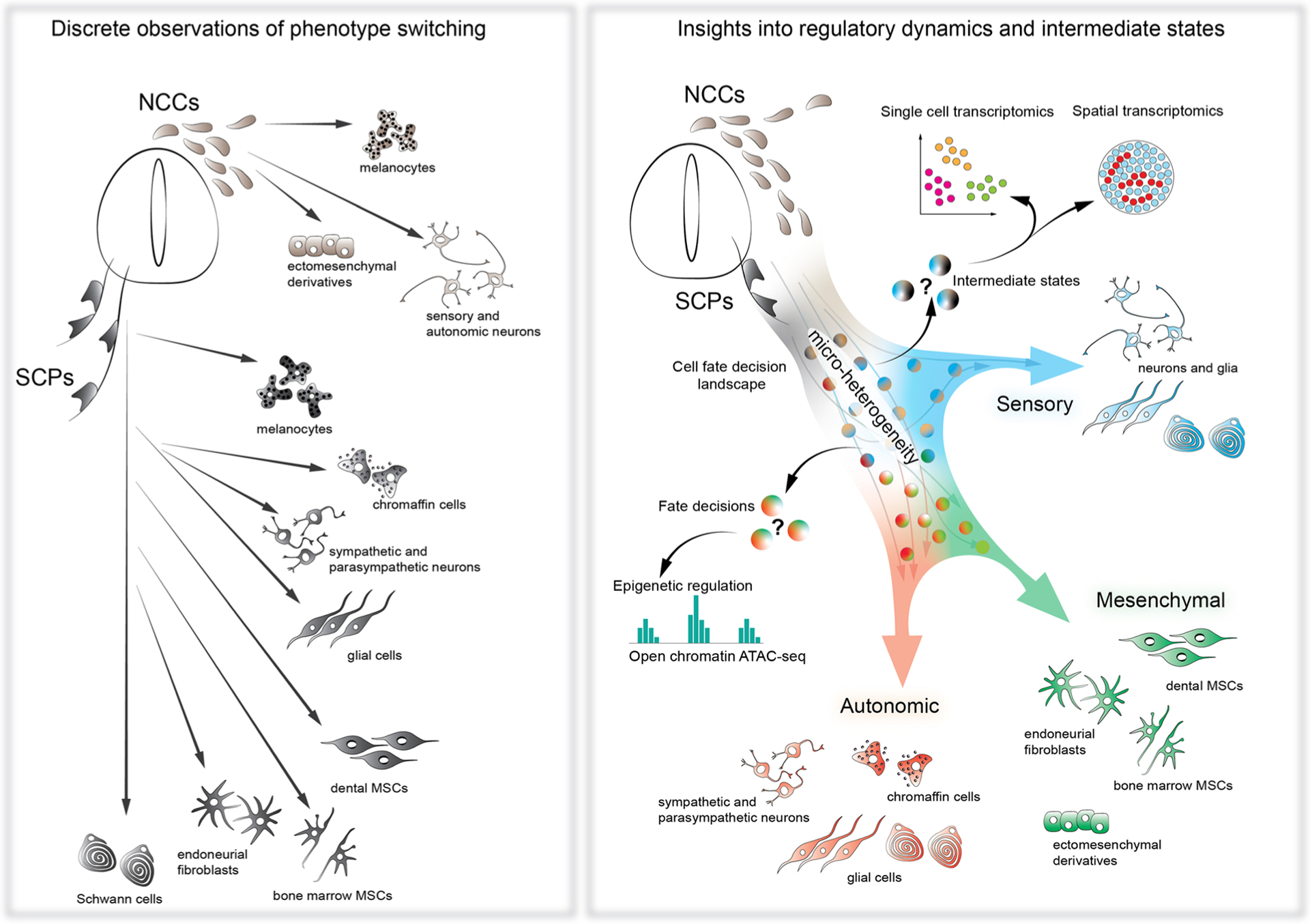
Single cell transcriptomics of the NCCs introduced the concepts of metastable states, micro-heterogeneity and pre-bifurcation dynamics into the old picture of neural crest differentiation. The historical and discrete observations of cell phenotype shifts in the population of the NCCs and crest-derived SCPs provided the information about general fate potential and some transitory steps in the developing neural crest population. However, the question of how the different fates are being specified remained largely elusive (**left scheme**). The single cell omics approaches provided a new vision of the mechanisms utilized by the NCCs during fate selection. The new insights resulted in the description of major transcriptional programs gradually changing under the control of intrinsic and extrinsic factors to generate specific fates in well-defined proportions. The older concepts of differentiation dynamics were aided by the discoveries of metastable intermediate states, pre-bifurcation dynamics and bias-dependent micro-heterogeneity (**right scheme**). NCCs – neural crest cells, SCPs – Schwann cell precursors, MSCs - mesenchymal stem cells.

We conclude this review with a remark about the transformative power of single cell transcriptomics, and multi-omics in general, towards understanding micro-heterogeneity that drives the logic of cell fate decisions in the neural crest lineage. Beyond being simply descriptive when it comes to gene expression and chromatin accessibility states, single cell technologies lean now towards predicting the causal interactions, elucidating hierarchical structures of gene regulatory networks, and inferring dynamic aspects from seemingly static snapshots of individual cells. The existing datasets now predict a myriad of functional interactions between multiple cell types and functional connections between gene regulatory networks with associated signal transduction mechanisms. However, it is still unknown how exactly the extrinsic signals are converted into intracellular biases directing the development of heterogeneity and fate choices in the lineage of NCCs (Fig. 6). Answering these questions requires integrated experimental platforms where cell states and lineages can be simultaneously probed. This will be achieved using single cell approaches that track clonal relationships, analysis of fine spatial heterogeneity, and multiplexed functional validations in vivo. In particular, the incorporation of clonal, spatial, and functional assays into the existing transcriptomic and epigenetic landscapes is a critical next step for understanding how communities of embryonic progenitor cell populations engage in interactive crosstalk to adaptively regulate cell fate specifications on the whole organism level.
